# Primary and Secondary Variants of Callous-Unemotional Traits in Community Youths: Differences in Anticipatory Fear

**DOI:** 10.3390/children11030359

**Published:** 2024-03-19

**Authors:** Yu Gao, Adrian Raine

**Affiliations:** 1 Department of Psychology, Brooklyn College, The City University of New York, 2900 Bedford Avenue, Brooklyn, NY 11210, USA; 2 The Graduate Center, The City University of New York, New York, NY 10016, USA; 3 Departments of Criminology, Psychiatry, and Psychology, University of Pennsylvania, Philadelphia, PA 19104, USA; araine@sas.upenn.edu

**Keywords:** psychopathy, development, fear, primary, secondary, psychophysiological

## Abstract

Callous-unemotional (CU) traits refer to a cluster of characteristics such as low empathy, lack of remorse, and insensitivity to the emotions of others, delineating a group of youth at high risk for severe antisocial behavior. Two variants—primary and secondary CU—have been theorized to have different underlying mechanisms, although mixed findings have been reported. The current study examined if the variants differ in their level of anticipatory fear in 92 youths from the community (mean age = 14.2 years, range = 12.3–16.4 years; 43.5% female). Participants completed a countdown task while their heart rate and skin conductance responses were recorded. Parents and youths completed the inventory of callous-unemotional traits and the child behavior checklist. Compared to the control group (low CU/low anxiety), the primary CU group (high CU/low anxiety) showed prolonged heart rate deceleration in anticipation of the impending aversive stimulus. The secondary CU group (high CU/high anxiety) did not differ from the other two groups on heart rate or skin conductance responses. This prolonged heart rate deceleration in the primary CU group is interpreted in the context of the passive vagal coping theory of antisocial behavior which hypothesizes that an over-engagement of the parasympathetic nervous system reduces the impact of a socializing punishment, which in turn predisposes individuals to antisocial behavior. Findings provide further support for the distinct etiology of two variants of CU traits.

## 1. Introduction

Callous-unemotional (CU) traits refer to a cluster of characteristics such as low empathy, lack of remorse, and insensitivity to others’ emotions, delineating some youth at elevated risk for severe antisocial behavior [1]. These traits are relatively stable from early childhood to young adulthood [2] and have significant genetic influences [3]. Empirical evidence has suggested that there are diverse etiological pathways to the development of primary and secondary variants of CU traits, consistent with the research examining multiple pathways to adult psychopathy. Specifically, it has been theorized that strongly heritable affective deficits contribute to the primary psychopathy variant, whereas the secondary psychopathy variant originates from adaptation to environmental adversity, such as early maltreatment or traumatic experiences (e.g., [4,5]. Similarly, the primary variant of CU is theorized to be characterized by low anxiety and arise from more heritable risk, whereas the secondary variant is characterized by high anxiety and maltreatment experiences [1,6,7]. Some initial evidence has even suggested that these two variants can be observed in children as young as three [8,9,10]. Given potential differences in etiologies and responsivities to treatment programs between the two variants [11], it is important to further understand the distinction between the two variants, as such knowledge may inform psychological assessments and interventions by helping in tailoring the approaches to therapy and support. 

The processing of fear is thought to be dysfunctional in children with CU traits. Moderate fear responses when anticipating impending aversive stimuli have been theorized to reinforce avoidance behavior to harmful events, whereas insufficient fear responses may underlie weak conscience development, which in turn contributes to psychopathic and externalizing behavior [12,13]. In addition, atypical fear processing may contribute to cognitive dysfunction that impairs socialization process in antisocial individuals [14]. Empirical evidence has supported the notion that fear deficits may be the core characteristics of CU. For example, compared to children with low CU, children with high CU traits showed reduced skin conductance responses to distress cues [15], lower levels of fear-potentiated startle [16], autonomic hypoarousal when viewing emotionally evocative scenes [17], reduced activation in the amygdala while processing fearful expressions [18], and lower ratings of arousal to unpleasant pictures [19]. This fearless temperament and deficits in processing stimuli may lead to their insufficient sensitivity to socializing cues, which in turn contribute to their disrupted development of conscience [20]. 

In this context of fear processing, anticipatory fear deficits have been reported in adolescents and adults with psychopathic traits, although findings have been mixed (e.g., [21,22,23]). Studies have used the countdown paradigm to investigate psychophysiological and behavioral responses to the impending aversive stimuli [21]. In a typical countdown paradigm, individuals are presented with numbers counting down on the screen, with the number zero resulting in an aversive stimulus such as a loud noise. Their psychophysiological responses, such as heart rate and skin conductance responses, are recorded to reflect their autonomic arousal during the anticipation period. In early studies conducted by Hare and colleagues, both psychopathic and non-psychopathic individuals showed heart rate deceleration following an initial acceleration. However, psychopathic individuals were found to show more heart rate acceleration relative to their non-psychopathic counterparts, although they did not differ on the heart rate deceleration following the acceleration period [24,25]. Some researchers reported larger heart rate acceleration to be associated with the callousness-disinhibition factor of the psychopathic traits in children aged 9–10 years from the community [23]. Others further showed that although psychopathic individuals showed larger heart rate acceleration when anticipating aversive tones, such acceleration was absent when they were able to prevent the tone [26]. It was thus hypothesized that the heart rate acceleration in anticipation of an aversive stimulus may reflect a successful internal coping response that reduces its impact as a premonitory cue to warn of the upcoming unpleasant stimuli [26,27]. However, a few studies have failed to find an association between psychopathy and heart rate changes [22,28,29]. 

Skin conductance activity has also been used to assess levels of anticipatory fear. Reduced skin conductance responses when anticipating impending threat have also been found in relationship to psychopathic traits. For example, one study found that 16-year-old boys with psychopathic traits showed significantly reduced skin conductance responses in anticipation of a 105-db white noise [21]. Another study found reduced anticipatory skin conductance responses in 9–10-year-old boys with the manipulative-deceitful factor of the psychopathic traits [23]. Such reduced skin conductance responding may index fearlessness and emotional deficits that underlie their propensity for antisocial behavior in psychopathic individuals [21]. Not all findings are consistent however, and in one recent study of adolescent male offenders, researchers failed to find a link between psychopathic traits and skin conductance responses when anticipating 90-dB white noises [22]. 

The mixed findings noted above on heart rate and skin conductance responses may be partly due to heterogeneity within children with high CU traits. In particular, atypical fear responses may be uniquely associated with the primary variant of psychopathy. In a recent review of 17 studies, researchers concluded that primary psychopathy is associated with attenuated fear levels whereas the experience of fear in secondary psychopathy is more intact [30]. Moreover, a more positive but not negative appraisal of the fear has been linked to primary psychopathy. It has been proposed that for the primary variant, the moral socialization process is disrupted due to deficits in emotion detection, arousal, and processing that has a biological basis [31]. Lack of intense and unpleasant emotional reactions (e.g., anxiety, fear, or guilty) to transgressions (e.g., aggressive behavior) may result in the absence or deficiency of the internal motivation system that is theorized to hinder aggressive or antisocial behavior [20,32]. Fear deficiency in primary psychopathy may be attributed to dysfunction in the limbic system, particularly the amygdala, which is a key brain region involved in fear processing [33]. In individuals with primary psychopathy, there are structural and functioning alternations in the limbic system, including the amygdala [34]. Therefore, deficiencies in this system are thought to contribute to the deficient processing of fear-related cues in these individuals, which may lead to increased rates of risk-taking and antisocial behavior. 

In contrast, children with the secondary variant of CU are theorized to show hyperarousal due to their oversensitivity to distress cues, which may make them overwhelmed by negative affect and disrupt their socialization process [35]. They are more likely to have been exposed to adverse environments at an early age [1], and as a result may develop emotional insensitivity, avoidance, and lack of empathy for others that function to provide psychological buffering to threatening situations [4]. Therefore, the reduced neural response to fear observed in primary psychopathy may not be seen in secondary psychopathy. The secondary variant may exhibit different neurocognitive correlates and patterns of brain activity. For example, they may show reduced activity in brain regions (e.g., superior temporal sulcus and thalamus) that are different from the areas affected in primary psychopathy [36]. These data further suggest that the fear response and underlying neurocognitive mechanisms in secondary psychopathy differ from those in primary psychopathy.

Taken together, studies have suggested that although both variants of CU have a core deficit in moral socialization, the primary pathway is underpinned by a heritable/dispositional affective deficit, whereas the secondary pathway is influenced by socio-environmental factors. The purpose of the study was to further examine the anticipatory fear deficits in CU traits by distinguishing the effects of primary and secondary variants of CU. Given that prior studies have suggested that primary psychopathy is associated with attenuated fear levels, and that the experience of fear is relatively intact in secondary psychopathy [30], we hypothesized that anticipatory fear deficits would be associated with the primary but not the secondary variant of CU. Although there are some differences on how to appropriately assess variants in prior studies, operationalizing these variants based on individuals’ anxiety scores is most common in studies with children and adolescents [1]. Therefore, in this study we explored the association between variants of CU and fear deficits by categorizing children with high CU into primary and secondary variants based on their level of anxiety. It was expected that compared to the low-risk group (low CU/low anxiety), the primary variant group (high CU/low anxiety) would show larger heart rate acceleration and lower skin conductance responses when anticipating aversive stimuli, whereas the secondary variant group (high CU/high anxiety) would not differ from the control group due to its relatively intact fear responses. 

## 2. Methods

### 2.1. Participants

Participants were recruited from a larger longitudinal study of community youths that examines connections between the psychophysiological, social, and environmental factors contributing to the development of emotional and behavioral problems in adolescence. In the original cohort (n = 340), 8–10-year-old children were recruited from the Brooklyn, New York area [37]. Within the study area, fliers were placed in recreation centers, libraries, churches, and other community centers, along with targeted mailings sent to parents with children within this age range. Children diagnosed with a psychiatric disorder, intellectual disability, or pervasive developmental disorder were excluded. A total of 92 adolescents participated in the current wave of data collection, comprised of 40 females and 52 males (age range: 13–16 years, mean age = 14.2, S.D. = 0.73). This high attrition rate resulted from an early determination of the project due to COVID-19 pandemic. Ethnic breakdown is as follows: African American (42.2%), Caucasian (21.1%), Hispanic/Latino (11.1%), Asian/Pacific Islander (3.3%), Native American (1.1%), and Other/Mixed (16.8%). Participating families received monetary compensation for their participation, and all procedures were approved by the Institutional Review Board of the university. 

Each child and their main caregiver participated in a 2 h testing session in the laboratory after they completed the youth assent and parental consent. The child then completed several computerized tasks, including the countdown task, while their physiological responses were recorded. The child and parent also filled out a questionnaire package in which they answered questions related to child’s personality traits and behavior, as well as demographic information such as age, race, and sex. Approximately half of the participants completed the questionnaires first and the other half completed the computerized tasks first. Families were debriefed after they completed all sessions.

### 2.2. Measures

Callous-Unemotional Traits. Participants completed the Inventory of Callous-Unemotional traits (ICU; [38]). Caregivers completed the caregiver version, while children completed the self-report version. Each version includes 24 items and ratings range from 0 (not at all true) to 3 (definitely true). For each participant, the greater score of the two reports was used, as recommended by the ICU and Antisocial Process Screening Device Manual [39]. Cronbach’s alpha for the total CU score in the current sample was 0.85 [40]. 

Anxiety. Caregivers completed the Child Behavior Checklist (CBCL; [41]) to rate the child’s behavior within the past 12 months. This rating scale includes 112 items, and items are rated on a 3-point scale: 0 = not true, 1 = sometimes true, and 2 = very true or often true. For the purposes of the present study, scores of the DSM-oriented anxiety subscale items were used in our analyses [42]. The validity of these DSM-oriented subscales in non-referred samples has been documented [43].

Following prior studies [44], group formation was conducted in three steps. First, a tertile split on the CU score was performed to identify those falling in the top and bottom third (n = 78). This tertile split resulted in a cut-off point of 34, which is consistent with the cut-off point used in [44] among a justice-involved adolescent sample. Second, among those retained, participants were separated according to whether they had higher anxiety score (high CU/high anxiety; secondary variant, n = 16) or lower anxiety score (high CU/low anxiety; primary variant, n = 13) based on the median split of the anxiety score. Median split was chosen to maximize the size of the non-overlapping groups. Finally, those with low CU and low anxiety were placed into the control group (n = 16).

### 2.3. Countdown Task

Participants completed a countdown task while sitting in front of a computer screen with headphones. Following prior studies [21,23], we included three signaled trials and two un-signaled trials. In each signaled trial, participants saw numbers (12–0, 1 s each) count down on the screen. They were informed in the beginning of the task that when the number reached 0, a loud noise (1 s burst of 105 dB white noise) would occur. For the un-signaled trials, they saw a blank screen, and with the same loud noise burst occurred without countdown. They did not know when the trial would start, or when the noise would occur. Only data from the signaled trials were included in the current study to evaluate the effects on anticipatory fear responses. After the countdown task, participants rated about the task on valance (1 = very unhappy, 5 = very happy) and arousal (1 = calm, 5 = intense) with the Self-Assessment Manikin [45]. They were also asked how they felt about the sound (1 = not at all unpleasant, 7 = extremely unpleasant). 

### 2.4. Psychophysiological Data Recording and Quantification

Heart Rate. A BIOPAC MP 150 system and AcqKnowledge 4.2 software (Biopac Systems, Inc., Goleta, CA, USA) were used to collect and analyze psychophysiological data. To record electrocardiogram (ECG) data, an ECG 100C amplifier was used, with two pre-jelled Ag-AgCl disposable vinyl electrodes placed at a modified Lead II configuration. The contact area was cleaned using rubbing alcohol before the electrodes were affixed. The ECG signal was converted to R-R intervals using the AcqKnowledge automated modified Pan-Tomkins QRS detector, after being visually inspected for artifacts. Heart rate (beats per minute; bpm) was computed based on the average of interbeat intervals (IBI; R-R wave intervals). Anticipatory heart rate responses were calculated as the differences between the mean heart rate during each of the 12 1 s intervals and the mean heart rate during the 5 s period preceding the trial. 

Skin Conductance Responses. A GSR 100C amplifier and TSD 203 were used to record electrodermal activity. Two 6-mm diameter silver/silver chloride electrodes were attached to the whorl of the distant phalanges of the first and second fingers of the non-dominant hand. The BIOPAC isotonic recording gel (GEL 101) was filled into the electrodes as the electrolyte medium. Next, double-sided adhesive collars were used to secure the electrodes on the fingers. To quantify skin conductance response amplitude, the peak-to-peak function in AcqKnowledge 4.2 (BIOPAC system, Inc., Goleta, CA, USA) was used. An elicited response was identified if the amplitude exceeded 0.05 μS during the 12 s countdown [21]. For trials that no response was detected, the magnitude would be zero (12.6% of the participants; [46]). Responses were averaged across the three signaled trials for each participant. To help attain normality, skin conductance responses were transformed using square root prior to any inferential statistical analyses [47].

### 2.5. Statistical Analysis 

All analyses were conducted using SPSS 29 (SPSS Inc., Chicago, IL, USA). All Likert scales were treated as continuous variables. To investigate the effects on heart rate change, repeated measures analysis of covariance (ANCOVA) was conducted with CU group (primary variant, secondary variant, and control) as a between-subject factor, time (12 s) as a within-subject factor, and sex and race as the covariate variables. For skin conductance responses, univariate ANCOVA was conducted with the CU group as the independent variable and the same covariate variables. Cohen’s d [48] and partial eta squared (0.01 indicates small, 0.06 indicates median, and 0.14 indicates large) were reported to indicate effect sizes.

## 3. Results

### 3.1. Descriptive Statistics

Table 1 presents demographic information, means, and standard deviations of the main variables for the three groups. Groups did not differ on age (F (2, 58) = 1.02, *p* = 0.37). There were more males and more Black/African Americans in the primary variant group, more females in the secondary variant group, and more Whites in the control group (for race, χ^2^ (4) = 14.92, *p* = 0.005; for sex, χ^2^ (2) = 9.76, *p* = 0.008). As expected, the secondary group had higher scores on anxiety than the other two groups (F (2, 58) = 8.35, *p* < 0.001). Since groups differed on sex and race, these two variables were controlled for in the following analyses.

### 3.2. Group Differences in Anticipatory Fear Responses

**Figure 1:**
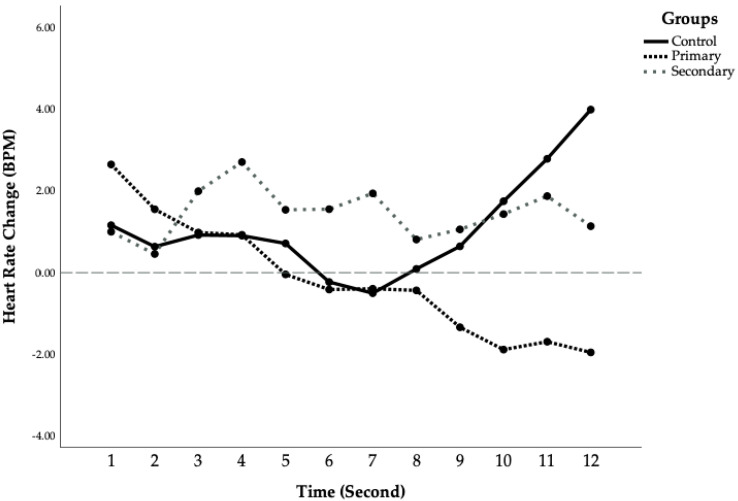
Mean heart rate change during signaled conditions for three groups.

Heart Rate and Skin Conductance Responses. For heart rate changes, repeated measures ANCOVA showed no significant main effect of time (F (11, 28) = 0.74, *p* = 0.69) or group (F (1, 38) = 0.81, *p* = 0.45), although the group by time interaction was marginally significant (F (22, 58) = 1.52, *p* = 0.05, η^2^ = 0.07). Further exploration showed that groups differed significantly during the last second before the noise onset (F (2,38) = 3.46, *p* = 0.04, η^2^ = 0.15). The primary CU group had significantly lower anticipatory heart rate than the control group (M_primary_ = −1.67, SD = 5.73, M_control_ = 3.82, SD = 6.11, *p* = 0.014, d = 0.93), whereas the secondary CU group did not differ from the other two groups (M_secondary_ = 1.08, SD = 5.34, *p* > 0.203). The mean second-by-second changes in heart rate during the anticipatory periods of signaled trials (loud noise expected) are presented in Figure 1. As can be seen in Figure 1, the primary CU group showed prolonged heart rate deceleration whereas the control group had more heart rate acceleration by the end of the countdown period. Finally, the groups did not differ on skin conductance responses, F (2, 39) = 0.39, *p* = 0.68 (see Table 1), although the primary CU group tended to have lower responding compared to both controls (d = −0.75) and the secondary CU group (d = −0.72).

Post-Task Ratings. The groups differed on the arousal rating of the task (F (2, 52) = 5.39, *p* = 0.007, η^2^ = 0.17): The primary variant group reported the task to be less intense than the other groups, whereas the two other groups did not differ on their arousal ratings. Finally, the groups did not differ on their ratings on the valance of the task (F (2, 52) = 0.68, *p* = 0.51), or the unpleasantness of the tone (F (2, 53) = 0.40, *p* = 0.67). (See Figure 1).

## 4. Discussion

The purpose of this study was to determine if anticipatory fear deficits were associated with the primary (but not the secondary) variants of CU traits. Using the typical countdown paradigm, we recorded heart rate and skin conductance responses when participants were expecting the impending aversive stimulus. Although the primary variant group did show different psychophysiological responses when anticipating the loud noise as compared to the controls, the direction of the difference was opposite to what we had expected. While the control group showed initial heart rate deceleration followed by a gradual acceleration, the primary CU group exhibited prolonged and increasing deceleration throughout the task. In addition, the primary CU group showed smaller, although not statistically significant, skin conductance responses than the other two groups. The secondary CU group did not differ significantly from the control group on either heart rate or skin conductance responses. Findings provide further support to the existence of multiple etiologies of CU, even in this non-referred sample. 

How can the heart deceleration in the primary CU group be best interpreted? Beat-by-beat heart rate changes are under the joint control of the sympathetic nervous system and the parasympathetic nervous system. While the sympathetic branch is often involved in the “fight or flight” response and acts to increase heart rate, the parasympathetic branch acts to decrease heart rate through the vagus nerve. We found that in the beginning of the countdown the primary variant group showed slight heart rate deceleration, similar to the low-risk control group. However, as the low-risk group showed heart rate acceleration in the second part of the countdown, the primary CU group demonstrated prolonged and increasing heart rate deceleration. Research has shown that when individuals are presented with novel stimuli, their heart rate decelerates or drops below baseline but then quickly returns to baseline [49]. The initial heart rate deceleration is theorized to be an orienting response, reflecting an increase in attention and active processing of sensory information [49]. However, empirical evidence has failed to support this theory by demonstrating that heart rate deceleration does not habituate when stimulus is repeatedly presented [50]. Alternatively, it has been argued that phasic heart rate deceleration seen in the primary CU group may be part of an early pre-attentive process of stimulus registration [51]. In contrast, the anticipatory heart rate acceleration seen in the control group may reflect a defensive inhibition of sensory input that helps the individual cope with the impending aversive stimuli by reducing the impact of the premonitory cues [52]. Overall, the lack of heart rate acceleration and prolonged heart rate deceleration seen in the primary variant group may be indicative of sustained attentional processing of the aversive stimuli, or alternatively ineffective coping in this group. A difficulty with this interpretation is that adult psychopaths show heart rate acceleration, not deceleration in anticipation of an aversive stimulus [25].

Other theoretical accounts of the findings need to be considered. In contrast to the sustained attention account, we would instead suggest as an alternative perspective that prolonged heart rate deceleration may reflect a momentary behavioral inhibition or freezing response that occurs while individuals are faced with a threatening situation [53]. According to the vagal passive coping theory of antisocial behavior, antisocial individuals may engage the parasympathetic nervous system in the face of impending and inescapable punishment (e.g., aversive tone), which in turn serves as a conservation/withdrawal mechanism to disengage the individual from the impending threat [54]. Prolonged heart rate deceleration may reflect an over-engagement of parasympathetic nervous system that results in immobilization and muscular relaxation that reduces the perception of pain [55]. This passive withdrawal is hypothesized to reduce the impact of a socializing punishment and thus predispose individuals to antisocial behavior [54]. Such freezing responses or passive withdrawal interpretation would be consistent with the significantly lower task arousal ratings reported by the primary CU group (see Table 1). While further research is needed to examine the specific mechanism underlying heart rate deceleration in the context of CU traits, the prolonged heart rate deceleration in primary CU children reflects at some level an atypical parasympathetic nervous system and vagal influence. 

In addition to heart rate, we also assess skin conductance activity as another autonomic indicator of anticipatory fear. In contrast to our a priori prediction, groups did not differ on skin conductance responses in anticipation of impending aversive stimuli. This null finding is in line with [22], who also failed to find psychopathic traits to be associated with skin conductance responses in anticipation to the signaled tone in a group of male adolescent offenders. However, it is noteworthy that although group differences were not statistically significant, they were in the predicted direction that the primary CU group had smaller responses than the other two groups, with effect sizes near to large (d = −0.72 to −0.75). As such, a larger sample size may have confirmed statistically significant differences. 

Limitations of the study need to be considered. A primary limitation is the small sample size, and thus low statistical power to detect significant effects such as the medium–large effect size for reduced skin conductance responding in the primary CU group. Nonetheless, the significant findings are considered moderate in magnitude. Our tertile split resulted in 31% of the sample being in the high CU group with a cut-off score of 34. This is in the high end of the range reported by [56], who found that 9–31% of school children have elevated CU traits. Nonetheless, the ICU cut-off score of 34 used in our study is consistent with [56] whose cut-off scores ranged from 21 to 46. Similarly, the percentages of primary (14%) and secondary (17%) CU groups in our sample were comparable to those reported in prior research (18–19%; [44]. In addition, due to low power we were not able to test potential mediation or moderation effects due to sex or ethnicity. Larger-scale studies are needed to detect whether males and females demonstrate different associations as some recent studies have reported psychophysiological abnormalities in males (but not females) with psychopathic traits [23,57]. Similarly, cross-cultural studies are needed to determine if similar neurobiological characteristics can be observed in different ethnic groups, since some studies have suggested that risk factors for CU traits may differ between Western and non-Western countries [58]. Finally, studies are needed to examine if our findings are generalizable to other age groups and other types of emotional responsivity measurements such as behavioral or self-report indices. It has been suggested that atypical emotional responsiveness in CU is more likely to be observed when studies use physiological measures and in older, adolescent samples [59], and this requires further resolution in future studies. 

In conclusion, our preliminary and initial findings suggest that anticipatory fear deficits may be a characteristic of the primary variant of CU but require replication on larger samples for further substantiation. This conclusion is broadly consistent with prior work that demonstrates distinct psychophysiological and behavioral characteristics between the primary and secondary variants in children with conduct problems [60,61]. Further delineation of the heterogeneity in children with high CU traits may help advance our understanding of the developmental pathways to childhood antisocial behavior and improve treatment of antisocial individuals, particularly those on the primary CU path. More experimental research in the future could further test the passive vagal coping theory of antisocial behavior by manipulating parasympathetic nervous system activity through either non-invasive transcutaneous auricular vagus nerve stimulation [62] or biofeedback training [63] to better understand the causal nature of the relationships between the parasympathetic nervous system, antisocial behavior, and primary CU traits.

## Figures and Tables

**Table 1:** Means (standard deviations) for demographics and main variables for overall sample and by group.

	Overall Sample (n = 92)	Primary Variant (n = 13)	Secondary Variant (n = 16)	Control (n = 16)	Group Comparisons
Male (%)	57%	83%	35%	45%	χ^2^ (2) = 9.76, *p* = 0.008
Age	14.20 (0.73)	14.77 (0.50)	14.46 (0.89)	14.47 (0.78)	F (2, 58) = 1.02, *p* = 0.37
Race	32% White, 42% Black, 26% others	0% White, 78% Black, 22% others	26% White, 32% Black, 42% others	41% White, 27% Black, 32% others	χ^2^ (4) = 14.92, *p* = 0.005
CU traits	30.25 (9.44)	38.78 (2.60)	42.76 (5.53)	19.41 (4.61)	F (2, 58) = 164.58, *p* < 0.001, η^2^ = 0.85, Secondary > Primary > Control
Anxiety	2.06 (2.71)	0.89 (1.45)	3.30 (3.10)	1.04 (1.17)	F (2, 58) = 8.35, *p* < 0.001, η^2^ = 0.73, Secondary > Primary = Control
Valance Rating	2.96 (0.93)	3.12 (1.00)	2.74 (0.81)	3.05 (1.31)	F (2, 52) = 0.68, *p* = 0.51
Arousal Rating	2.88 (1.21)	2.12 (1.17)	3.11 (1.24)	3.31 (1.06)	F (2, 52) = 5.39, *p* = 0.007, η^2^ = 0.17, Primary < Secondary = Control
Sound Unpleasantness Rating	4.18 (1.73)	3.94 (1.89)	4.45 (1.76)	4.37 (1.83)	F (2, 53) = 0.40, *p* = 0.67
Skin Conductance Responses	0.61 (0.73)	0.28 (0.32)	0.73 (0.79)	0.64 (0.58)	F (2, 39) = 0.39, *p* = 0.68

## Data Availability

Data will be made available upon reasonable request. The data are not publicly available due to specific ethical and privacy considerations.
